# ACCORD: an assessment tool to determine the orientation of homodimeric coiled-coils

**DOI:** 10.1038/srep43318

**Published:** 2017-03-07

**Authors:** Byeong-Won Kim, Yang Ouk Jung, Min Kyung Kim, Do Hoon Kwon, Si Hoon Park, Jun Hoe Kim, Yong-Boo Kuk, Sun-Joo Oh, Leehyeon Kim, Bong Heon Kim, Woo Seok Yang, Hyun Kyu Song

**Affiliations:** 1 Division of Life Sciences, Korea University, 145 Anam-ro, Seongbuk-gu, Seoul 02841, Korea; 2 Center for Molecular Dynamics and Spectroscopy, Institute of Basic Science, Seoul 02841, Korea

## Abstract

The coiled-coil (CC) domain is a very important structural unit of proteins that plays critical roles in various biological functions. The major oligomeric state of CCs is a dimer, which can be either parallel or antiparallel. The orientation of each α-helix in a CC domain is critical for the molecular function of CC-containing proteins, but cannot be determined easily by sequence-based prediction. We developed a biochemical method for assessing differences between parallel and antiparallel CC homodimers and named it ACCORD (Assessment tool for homodimeric Coiled-Coil ORientation Decision). To validate this technique, we applied it to 15 different CC proteins with known structures, and the ACCORD results identified these proteins well, especially with long CCs. Furthermore, ACCORD was able to accurately determine the orientation of a CC domain of unknown directionality that was subsequently confirmed by X-ray crystallography and small angle X-ray scattering. Thus, ACCORD can be used as a tool to determine CC directionality to supplement the results of *in silico* prediction.

A coiled-coil domain (CCD) consists of two or more α-helices that are twisted around each other to form a superhelical structure^1^. Coiled-coils (CCs) have a heptad repeat pattern (a-b-c-d-e-f-g)_n_, in which the *a* and *d* positions are usually occupied by hydrophobic residues. Based on this sequence feature, CCs are easily detected by prediction programs or servers such as COILS^2^, Marcoil^3^, Paircoil2 (ref. 4), and SOCKET^5^. However, they are very versatile structural domains that can adopt many different structures^1^. They can form different oligomeric complexes, and the helix orientation can either be parallel or antiparallel. Because of these characteristics, CCs participate in diverse protein-protein interactions that are involved in many biological functions and components such as fibrous filament formation^6^, gene regulation^7^,^8^, development of cartilage and bone^9^, membrane channels^10^, protein degradation^11^,^12^, protein trafficking^13^,^14^,^15^, and molecular chaperones^16^. To understand the detailed molecular functions of CC-containing proteins, it is absolutely necessary to determine the relative orientation of each coil, because the functional domain outside of a parallel CCD dimer must be in close proximity, whereas that outside an antiparallel CCD is located further away. Many programs and servers have been developed to determine the oligomeric state of CCDs; these include SCORER 2.0 (ref. 17), PrOCoil^18^, Multicoil2 (ref. 19), and LOGICOIL^20^. However, none of these programs adequately discriminates between the parallel and antiparallel orientations.

As an alternative to *in silico* prediction of CC orientation, the introduction of a cysteine residue at an appropriate site in a helix can be used to determine the CC orientation by analysing the resulting intermolecular disulphide bridge^21^,^22^. However, although strategies to rapidly evaluate sequence-stability relationships in the parallel coiled-coil motif have been reported^23^,^24^, this approach has the following limitations: first, a cysteine mutation in a CCD often cannot form a proper disulphide bond because of incorrect positioning, overall structural perturbation, and/or oxidation of the sulphur atom^25^,^26^,^27^; and second, the CC molecule itself is sometimes not properly assembled in a heterologous expression system^28^ and shows low solubility (Supplementary Fig. 1).

To overcome the above limitations, we developed a new biochemical technique using a fusion tag, which is an appropriately spaced and oriented molecule that enhances the solubility of CC proteins, to assess the orientation of the CC dimer.

## Results

### Stringent starvation protein B (SspB) is an appropriate fusion tag

**Figure 1:**
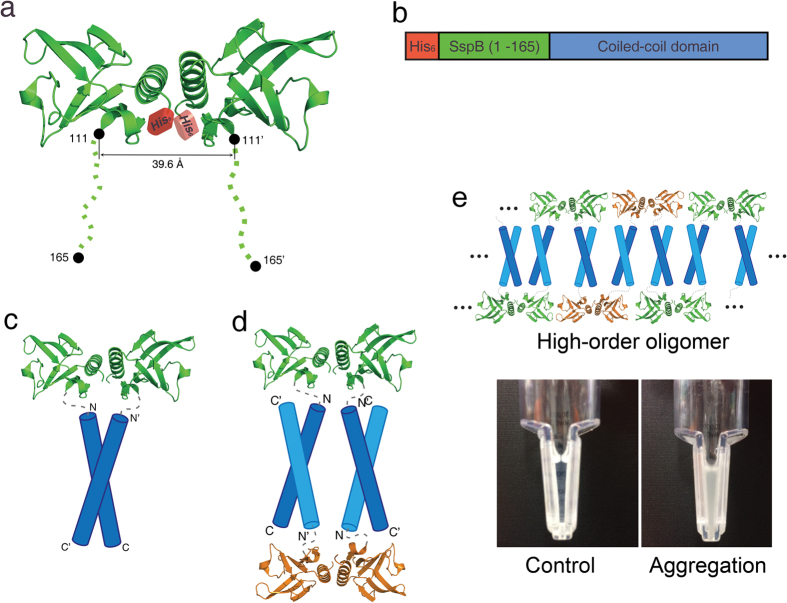
Design of the stringent starvation protein B (SspB)-fusion protein. (**a**) Overall structure of the SspB protein. The tail residues from 112 to 165, shown in dots, were not modelled because of their flexibility. The distance between Asp111 and Asp111′ is approximately 40 Å. The N-terminal hexa-histidine tag is shown as a red block. (**b**) Domain architecture of the SspB-CCD fusion protein. The N-terminal hexa-histidine tag, full-length SspB (residues 1 to 165), and target coiled-coil domain (CCD) are coloured red, green, and blue, respectively. (**c**) Schematic model of a dimer of SspB fused with a parallel CCD. The N- and C-termini of the CCs are indicated. (**d**) Schematic model of a possible arrangement of a tetramer of SspB fused with an antiparallel CCD. The N- and C-termini of the CCs are indicated. (**e**) A possible arrangement of higher-order oligomers of SspB fused with an antiparallel CCD and protein aggregation during concentration of the sample (left: soluble control protein; right: aggregation of higher-order oligomers).

As a fusion tag, we used SspB, which is an adaptor protein that delivers ssrA-tagged proteins to the ClpXP degradation machine in *Escherichia coli*^29^,^30^. SspB is a dimeric protein with two long and flexible C-terminal tails, both of which extend in the same direction (Fig. 1a)^31^,^32^,^33^. For the purpose of purification, hexa-histidine residues were added to the N-terminus, and the target CCD was attached to the C-terminus of full-length SspB (residues 1–165, with a 55-amino acid residue long C-terminal tail) (Fig. 1b). The C-terminal tail has enough degrees of freedom to accommodate each coil in the CCD. The idea is simple and is as follows: If the CC dimer is oriented in a parallel manner, the SspB-CC fusion protein behaves as a dimer in solution, as shown in Fig. 1c; however, if the CC dimer is oriented in an antiparallel manner, the SspB-CC fusion protein behaves as a soluble tetramer (Fig. 1d) or forms higher-order oligomers and/or aggregates (Fig. 1e).

### Size-exclusion chromatography with multi-angle light scattering (SEC-MALS) analyses of parallel or antiparallel CC proteins

**Figure 2:**
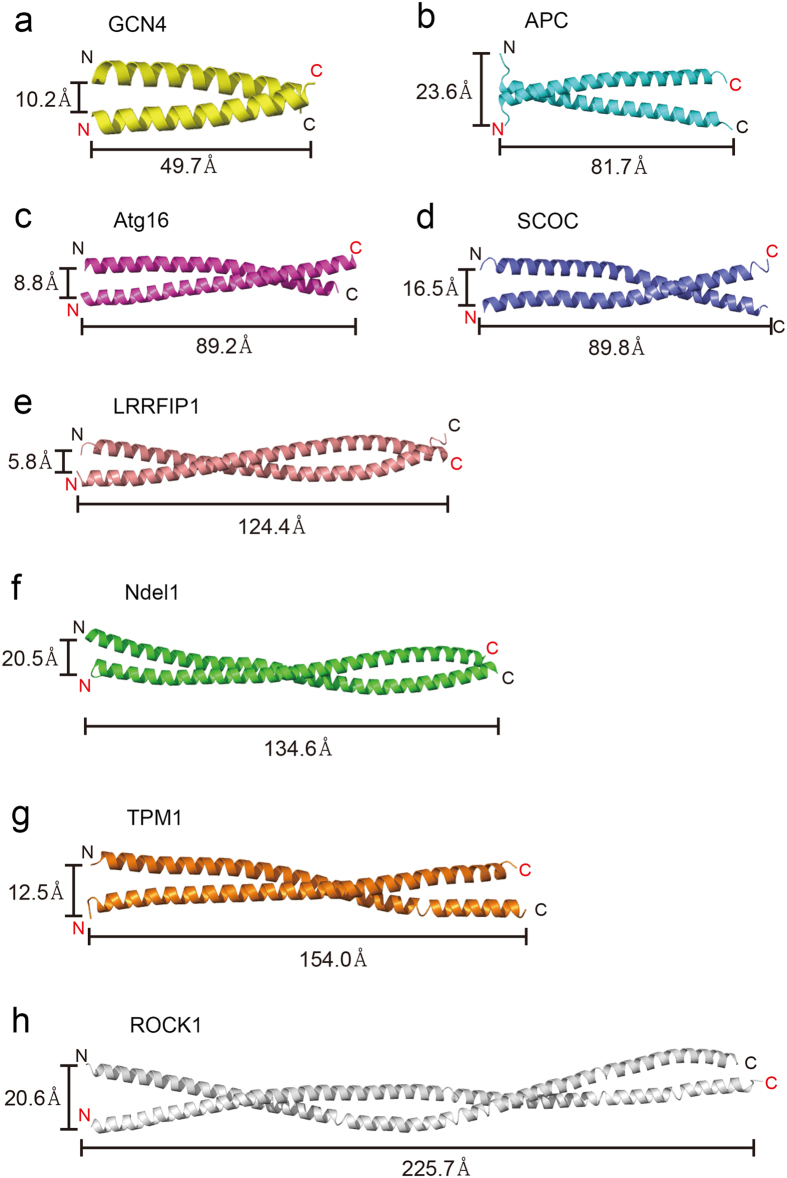
Structures of parallel coiled-coil domains (CCDs). (**a**) GCN4 from *Saccharomyces cerevisiae*. (**b**) APC, adenomatous polyposis coli protein from *Home sapiens*. (**c**) Atg16, autophagy protein 16 from *S. cerevisiae*. (**d**) SCOC, short coiled-coil protein from *H. sapiens*. (**e**) LRRFIP1, leucine-rich repeat flightless-interacting protein 1 from *H. sapiens*. (**f**) Ndel1, nuclear distribution protein nudE-like 1 from *Rattus norvegicus*. (**g**) TPM1, tropomyosin α-1 chain from *Gallus gallus*. (**h**) ROCK1, Rho-associated coiled-coil containing protein kinase 1 from *H. sapiens*. The length of each CCD and the Cα distance between the two N-terminal residues are provided.

**Figure 3:**
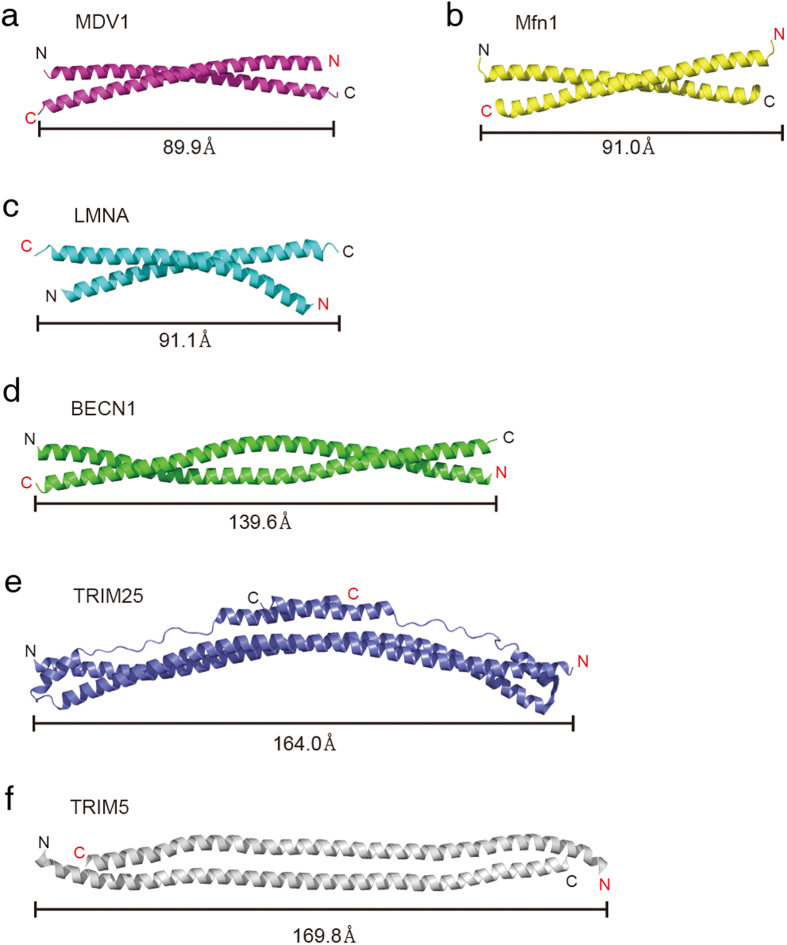
Structures of antiparallel coiled-coil domains (CCDs). (**a**) MDV1, mitochondrial division protein 1. (**b**) Mfn1, Mitofusin-1. (**c**) LMNA, prelamin-A/C. (**d**) BECN1, beclin-1. (**e**) TRIM25, E3 ubiquitin/ISG15 ligase tripartite motif-containing protein 25. (**f**) TRIM5, tripartite motif-containing protein 5. The length of each CCD is provided.

**Figure 4:**
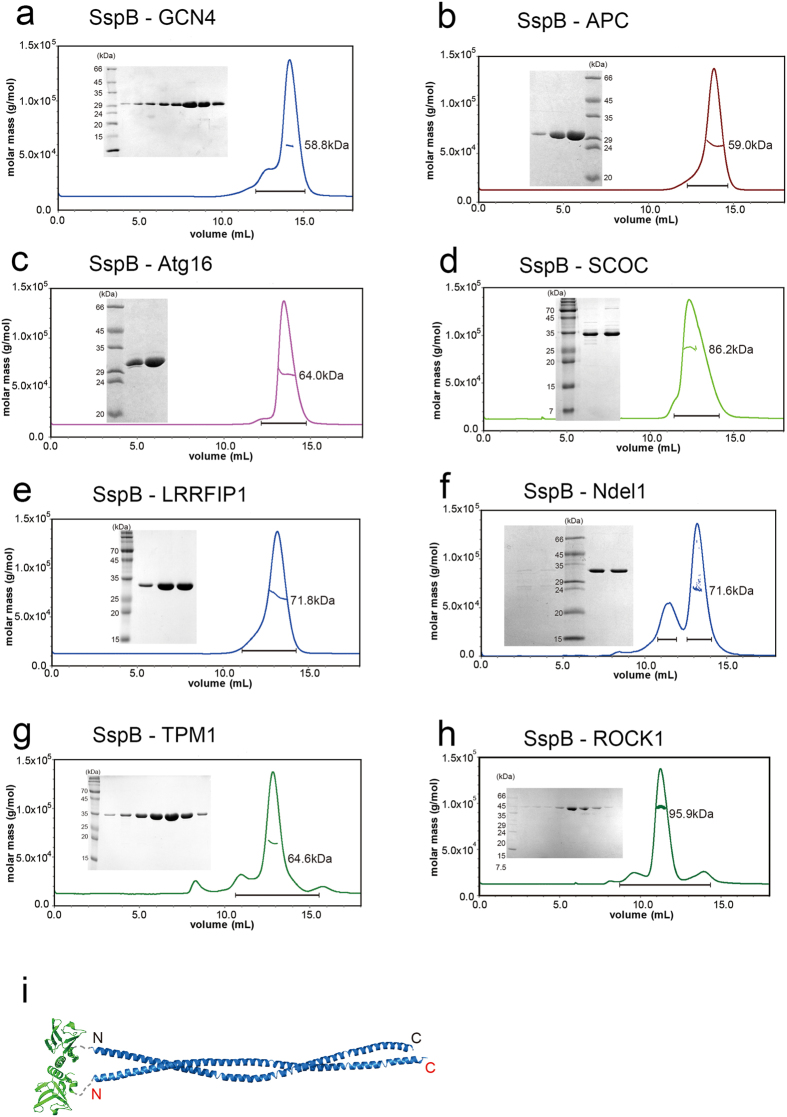
ACCORD results for parallel coiled-coils (CCs). Size-exclusion chromatography with multi-angle light scattering data from (**a**) SspB-GCN4, (**b**) SspB-APC, (**c**) SspB-Atg16, (**d**) SspB-SCOC, (**e**) SspB-LRRFIP1, (**f**) SspB-Ndel1, (**g**) SspB-TPM1, and (**h**) SspB-ROCK1 fusion proteins. Insets show the results of sodium dodecyl sulphate-polyacrylamide gel electrophoresis of the chromatography fractions. (**i**) Schematic model of the SspB-ROCK1 dimer. All parallel CCDs fused with the SspB protein behaved as dimers in solution.

**Figure 5:**
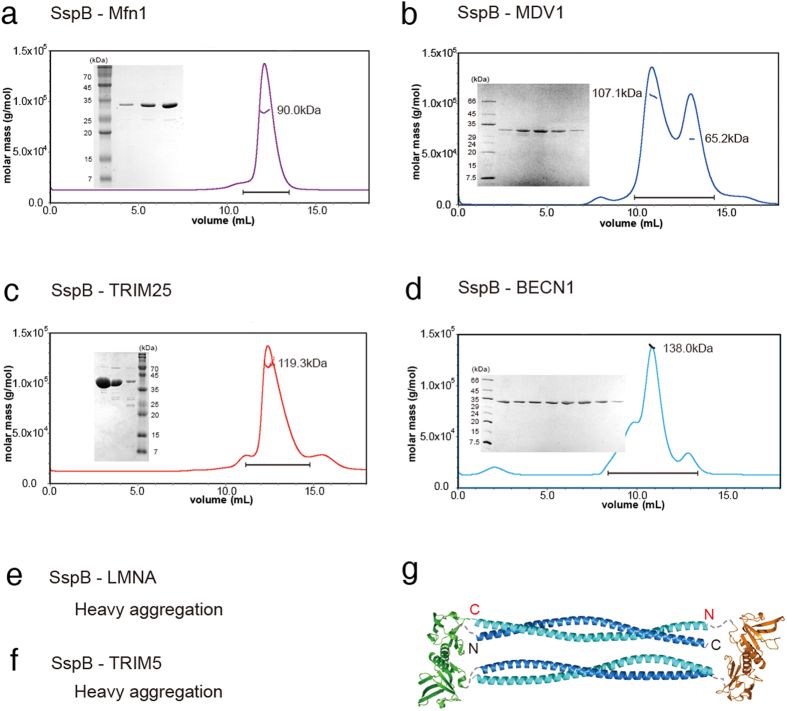
ACCORD results for antiparallel coiled-coils. Size-exclusion chromatography with multi-angle light scattering (SEC-MALS) data from (**a**) stringent starvation protein B (SspB)-Mfn1, (**b**) SspB-MDV1, (**c**) SspB-TRIM25, and (**d**) SspB-BECN1 fusion proteins. Insets shows the results of sodium dodecyl sulphate-polyacrylamide gel electrophoresis of the chromatography fractions. SEC-MALS analysis was not possible for (**e**) SspB-LMNA and (**f**) SspB-TRIM5 because of their significant precipitation. (**g**) Schematic model of the SspB-BECN1 tetramer. Antiparallel CCDs fused with the SspB protein behaved as tetramers or heavy aggregates in solution.

To validate this approach, we selected several parallel and antiparallel CC proteins whose structures have already been solved. The length of the CCDs varied from relatively short (49.7 Å) to long (225.7 Å; Figs 2a–h and 3a–f). In total, 14 parallel and antiparallel CC proteins were cloned into expression vectors, and the expressed fusion proteins were analysed by SEC-MALS. Molecular weight (MW) determination by MALS is critical, since dimeric CC proteins usually elute earlier than expected in SEC because of their elongated shapes. Intriguingly, the results were significantly different between parallel and antiparallel CCDs. Parallel CC proteins (GCN4, APC, Atg16, SCOC, LRRFIP1, Ndel1, TPM1, and ROCK1; see the legend in Table 1 for the full names of these proteins) were clearly dimeric in solution (Fig. 4a–h). When fused with SspB, the CCD of ROCK1 kinase, which is very long (225.7 Å)^34^, behaves as a 95-kDa dimer in solution (Fig. 4h,i). It must be noted that the distance between the two N-termini of parallel CCDs was in the range of 5.8–23.6 Å, which might fit well within the flexible C-terminal tails of SspB. In rare cases, the presence of a different oligomeric state was detected, but the major peak always corresponded to a dimer of parallel CCDs (Fig. 4f,g). This is an extremely good result compared to those of other prediction methods. LOGICOIL is very powerful for analysing the oligomeric state and orientation of CCDs^20^; however, its prediction of the orientation of dimeric CCs was not sufficiently accurate (Table 1). The first predictions were incorrect for two out of eight parallel CCDs. A bigger problem was that the prediction of antiparallel CCDs was not accurate at all. Only one out of six predictions was accurate in the case of antiparallel CCDs; the others were incorrect oligomeric structure predictions (Table 1). The antiparallel CC proteins (MDV1, Mfn1, LMNA, BECN1, TRIM25, and TRIM5; see the legend in Table 1 for the full names of these proteins) usually formed aggregates or tetramers in solution (Fig. 5a–f). The soluble tetramer is likely to be arranged as shown in Fig. 5g, but it is difficult to produce a single prediction model for higher-order oligomers (Fig. 1e). For some CCDs fused with SspB, it was difficult to obtain SEC-MALS data because the CCDs were prone to aggregation, and this phenomenon was most apparent with antiparallel CCDs. The short antiparallel CCD, MDV1, showed a significant proportion to be dimers, although the main fraction corresponded to a tetramer (Fig. 5b). Therefore, the orientation of a CCD can be easily assessed by analysing the oligomeric nature of SspB-fused CCDs. We have termed this method ACCORD.

### Application of ACCORD to unknown CC orientations

**Figure 6:**
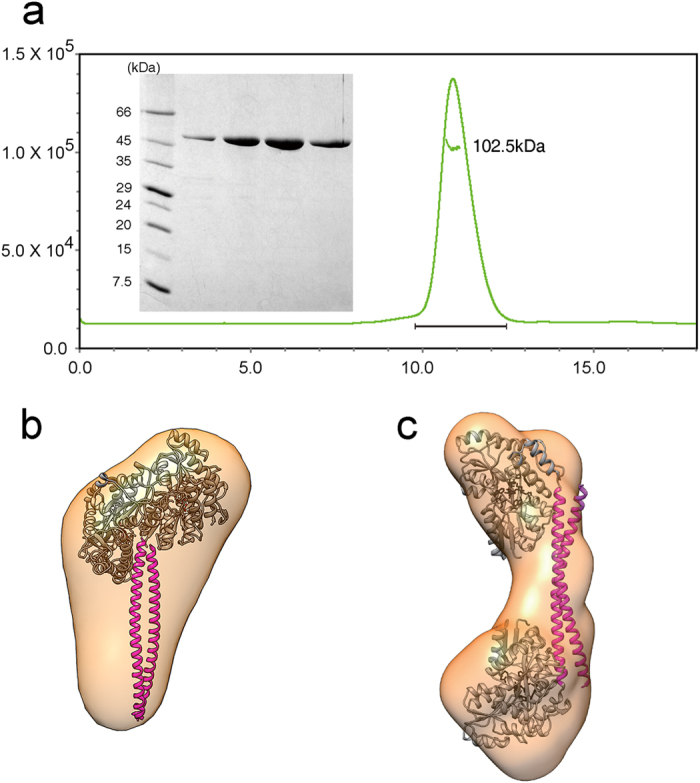
Validation of ACCORD results. (**a**) Size-exclusion chromatography with multi-angle light scattering (SEC-MALS) data showed that SspB-NDP52 is a dimer in solution, suggesting that the coiled-coil domain (CCD) of NDP52 is oriented in a parallel manner. Inset shows the results of SDS-PAGE of the chromatography fractions. (**b**) *Ab initio* envelope using SAXS data superimposed onto two MBP molecules (PDB ID: 3DM0) and a parallel CCD of Atg16 (PDB ID: 3A7O), which might have similar CC lengths based on the numbers of residues. (**c**) Molecular envelope of MBP-MDV1. The high-resolution crystal structure of MBP-MDV1 (Supplementary Fig. 2) was fitted into the low resolution molecular envelope generated from SAXS data.

Next, we applied the ACCORD method to CCDs with unknown orientations. NDP52, a dimeric autophagy receptor, consists of multiple domains, including a central CCD^35^,^36^. The CCD of NDP52 fused with SspB was subjected to SEC-MALS and was observed as a clear dimer in solution (Fig. 6a). Therefore, NDP52 was judged to be a parallel dimer based on our ACCORD method. To confirm the directionality of the CCs of NDP52, we attempted to determine the structure of the protein, but it did not crystallize successfully. Therefore, we used small angle X-ray scattering (SAXS) instead. If the fusion partner is a bigger monomeric protein, the molecular envelope is either Y-shaped (in the case of a parallel CC; Fig. 6b) or dumbbell-shaped (in the case of an antiparallel CC; Fig. 6c). The maltose binding protein (MBP) fused with the CCD of NDP52 shows a clear Y-shaped molecular envelope by SAXS (Fig. 6b), verifying that the CCD of NDP52 is parallel. In order to test an antiparallel CCD, we performed X-ray crystallography (Supplementary Fig. 2 and Supplementary Table 1) and SAXS of the MBP-MDV1 fusion protein (Supplementary Figs 3 and 4 and Supplementary Table 2). The results clearly showed that it has a dumbbell-shaped, rather than a Y-shaped, molecular envelope (Fig. 6c). These results were all consistent with those from our ACCORD method.

## Discussion

Given the central role of CCs in protein structure, the ability to easily determine the orientation of the helices in a CCD is important. Undoubtedly, a method to determine the orientation of CCD helices inside a cell would be really useful. There are several approaches to determine the global topology of *E. coli* membrane proteins using fusion techniques with reporter proteins, alkaline phosphatase, and green fluorescence protein^37^, and to establish the networks of basic region leucine zipper protein-protein interactions using a fluorescence resonance energy transfer-based assay^38^. These techniques are somewhat similar to ACCORD, in terms of using a fusion protein; however, their purposes are completely different from that of the ACCORD technique. Furthermore, proteome-wide approaches have limitations such as false positives; thus, *in vitro* experimentation is necessary to determine the orientation of CCs in a particular protein.

CCs are very structurally diverse and can exist in many different oligomeric states, including dimers, trimers, tetramers, and others. The majority of CCs form dimers, and the ACCORD technique was applied to homodimeric CCs because the fusion tag, SspB, is a homodimer. The use of SspB with a long C-terminal tail has pros and cons. The long and flexible tail acts as a linker that provides structural plasticity to accommodate structurally diverse CCs. However, our preliminary analysis suggests that it might be difficult to use the ACCORD technique to assess antiparallel CCs that are relatively short. For example, the ACCORD result for human MYO10 CC (51 amino acid residues and 47.1 Å; expected as a tetramer) showed that it was a dimer in solution (Supplementary Fig. 5a,b), and we speculate that it forms a dimer with the orientation shown in Supplementary Fig. 5c. Although the current version of the SspB fusion tag has some limitations, we are in the process of improving it for greater assessment accuracy.

Therefore, the ACCORD method can be used as a tool to determine homodimeric CC directionality to supplement *in silico* predictions that are inaccurate because the energy difference between the correct and incorrect directionalities of CCs is not large enough for discrimination between them. For a definitive answer, a three-dimensional structure determination by X-ray crystallography or nuclear magnetic resonance spectroscopy is best, but these methods are labour intensive and are sometimes technically infeasible because of the lack of diffracting crystals or because of the high molecular weight of a CC sample. The ACCORD approach is straightforward and is a good complement to *in silico* prediction methods to determine the CC orientation in a protein. By combining the ACCORD method and currently available prediction methods, the molecular function of target CC proteins can be interpreted with confidence.

## Methods

### DNA manipulation

Full-length *sspB* was amplified from *E. coli* genomic DNA by PCR and cloned into the NcoI and BamHI restriction sites of a modified pET vector (containing tobacco etch virus and thrombin cleavage sites) to construct an N-terminal hexa-histidine-tagged protein (hereafter, His_6_-SspB). The gene fragments GCN4 (249–281) and MDV1 (231–300) from the genomic DNA of *Saccharomyces cerevisiae* and APC (2–55), SCOC (78–159), LRRFIP1 (162–249), Beclin-1 (174–266), ROCK1 (535–709), and NDP52 (141–334) from the cDNA of *Homo sapiens* were amplified using primers containing restriction enzyme sites (BamHI at the 5′-end and EcoRI or XhoI at the 3′-end; NEB). The gBlock gene fragments Ndel1 (8–99), MYO10 (883–933), TPM1 (1–98), Mfn1 (600–735), LMNA (328–398), TRIM25 (194–356), and TRIM5 (133–241) were synthesized using primers containing the above-mentioned restriction enzyme sites (Integrated DNA Technologies). Sequences of the CCD proteins used in this study are provided in Supplementary Table 3. Genes for the CCDs were ligated into the His_6_-SspB vector. The gene fragments MDV1 (231–300) and NDP52 (197–270) were also ligated into a modified pMAL vector with an N-terminal hexa-histidine sequence and several point mutations to facilitate crystallization^39^. The resulting plasmids were transformed into *E. coli* BL21(DE3) cells.

### Protein expression and purification

His-tagged fusion protein expression was induced by the addition of 0.5 mM isopropyl-β-D-thiogalactoside at 291 K for 20 h. Cells were harvested by centrifugation and resuspended in buffer A (50 mM Tris, pH 8.0, and 0.5 mM tris(2-carboxyethyl)phosphine) containing 300 mM NaCl. After sonication, the cell lysate was loaded onto a HisTrap column (GE Healthcare) and then eluted by gradient purification with buffer A containing 100 mM NaCl and 500 mM imidazole. Eluted proteins were concentrated by ultrafiltration (Amicon Ultra 30 K NMWL, Millipore) and loaded onto a Superdex 200 10/300 GL column (GE Healthcare) equilibrated with buffer A containing 200 mM NaCl.

Cells expressing the MBP-tagged fusion proteins were harvested by centrifugation and resuspended in buffer A containing 100 mM NaCl and 1 mM EDTA. After sonication, the cell lysate was applied to a column containing amylose resin (NEB) and collected by gravity flow. The beads were washed with 10 column volumes of buffer A, and then the protein was eluted with buffer A supplemented with 10 mM maltose. The eluted sample was further purified using a Q-FF column (GE Healthcare). Finally, the fusion proteins were loaded onto a HiLoad 16/600 Superdex 200 column (GE Healthcare) pre-equilibrated with buffer A containing 100 mM NaCl.

### SEC-MALS

SEC-MALS experiments were performed using a fast protein liquid chromatography system (GE Healthcare) connected to a Wyatt MiniDAWN TREOS MALS instrument and Wyatt Optilab rEX differential refractometer. The column and buffer used were the same as those used in the final purification step. Ovalbumin was used as the isotropic scatterer for detector normalization. Light scattering from each sample (3–5 mg/ml, 0.5 ml) was measured and analysed using ASTRA V software (Wyatt).

### Crystallization and structure determination

Purified MBP-MDV1 fusion protein was crystallized at 295 K using the hanging drop vapour diffusion method and mixing an equal volume of the protein and a reservoir solution containing 100 mM sodium acetate, pH 4.5, 25% (w/v) polyethylene glycol 3350, 100 mM CaCl_2_, and 1.3–1.7 M sodium formate. Crystals were flash-frozen with the reservoir solution containing 20% (v/v) glycerol in a nitrogen stream at 100 K. Native MBP-MDV1 data were collected at beamline 5 C of the Pohang Accelerator Laboratory (PAL), Korea. Diffraction data were indexed, integrated, and scaled using HKL2000 software^40^. The structure was determined by molecular replacement using the MBP mutant structure as a search model^39^. Statistics for data collection and refinement are provided in Supplementary Table 1.

### SAXS

Solutions of the MBP-NDP52 and MBP-MDV1 fusion proteins were prepared in buffer A containing 100 mM NaCl. The concentration of MBP-NDP52 was 2.6 mg/ml, and that of MBP-MDV1 was 9.8 mg/ml. Scattering data of MBP-NDP52 and MBP-MDV1 were collected at beamline BL-10C of the Photon Factory, Japan, and at beamline 4 C of PAL, respectively. Details of experimental parameters are shown in Supplementary Table 2. Briefly, scattering images from the proteins at various concentrations were reduced to two-dimensional (2D) data by circular integration. A preliminary analysis of these 2D data using PRIMUS (ATSAS program) provided the radius of gyration (R_g_), Porod volume, and experimental molecular weight^41^. *Ab initio* modelling and averaging of these models were performed using DAMMIF and DAMAVER, respectively. Rigid body modelling of the crystallographic structure on dummy atom models was computed using the Situs program package^42^.

### Protein structure modeling

All protein models were generated using the crystal structures of SspB (PDB accession code: 1OX8)^32^ and the CCDs of MDV1 (PDB accession code: 2XU6)^43^, Beclin-1 (PDB accession code: 3Q8T)^44^, Atg16 (PDB accession code: 3A7O), and ROCK1 (PDB accession code: 3O0Z)^34^. All figures for structures were generated using PyMOL (http://www.pymol.org).

## Additional Information

**Accession code:** Atomic coordinates and structure factor files for MBP-MDV1 have been deposited in the Protein Data Bank with the accession code 5JST.

**How to cite this article**: Kim, B.-W. *et al*. ACCORD: an assessment tool to determine the orientation of homodimeric coiled-coils. *Sci. Rep.* **7**, 43318; doi: 10.1038/srep43318 (2017).

**Publisher's note:** Springer Nature remains neutral with regard to jurisdictional claims in published maps and institutional affiliations.

## Supplementary Material

Supplementary Information

## Figures and Tables

**Table 1:** Summary of ACCORD results in comparison with the results of LOGICOIL prediction.

Gene (*Origin*)	Residues	CC length (Å)	1^st^ prediction* (score)	2^nd^ prediction* (score)	MW of Fusion protein/CC (kDa)**	MALS (kDa)	Oligomer (ACCORD)	PDB (ref)	
SspB (*E. coli*)	M1–K165	—	—	—	18.3	—	—	1OX8 (ref. 32)	
Parallel (Para)									
GCN4 (*S. cerevisiae*)	R249–R281	49.7	Para dimer (1.25)	Anti dimer (0.97)	23.4/4.0	58.8	Dimer	2ZTA^7^	
APC (*H. sapiens*)	A2–I55	81.7	Para dimer (1.32)	Trimer (1.19)	25.5/6.1	59.0	Dimer	1DEB^45^	
Atg16 (*S. cerevisiae*)	V58–R130	89.2	Para dimer (1.37)	Anti dimer (0.89)	27.8/8.4	64.0	Dimer	3A7O^***^	
SCOC (*H. sapiens*)	M78–K159	89.8	Trimer (1.11)	Anti dimer (1.03)	28.8/9.4	86.2	Dimer	4BWD^46^	
LRRFIP1 (*H. sapiens*)	D162–E249	124.4	Para dimer (1.14)	Anti dimer (0.97)	30.0/10.6	71.8	Dimer	4H22 (ref. 47)	
Ndel1 (*R. norvegicus*)	D8–D99	134.6	Para dimer (1.14)	Anti dimer (0.97)	30.4/11.0	70.0	Dimer	2V71 (ref. 48)	
TPM1 (*G. gallus*)	M1–E98	154.0	Para dimer (1.05)	Tetramer (1.04)	30.6/11.2	64.6	Dimer	3U1A^49^	
ROCK1 (*H. sapiens*)	L535–K709	225.7	Tetramer (1.12)	Anti dimer (1.02)	40.0/20.6	95.9	Dimer	3O0Z^34^	
Antiparallel (Anti)									
MDV1 (*S. cerevisiae*)	Q231–G300	89.9	Tetramer (1.08)	Anti dimer (1.03)	27.6/8.2	65.2/107.1	Dimer/tetramer	2XU6 (ref. 43)	
Mfn1 (*M. musculus*)	F660–H735	91.0	Tetramer (1.21)	Anti dimer (1.0)	28.2/8.8	90.0	Tetramer	1T3J^50^	
LMNA (*H. sapiens*)	A328–S398	91.1	Tetramer (1.11)	Para dimer (1.09)	27.9/8.5	—	Aggregation	2XV5 (ref. 51)	
BECN1 (*H. sapiens*)	E174–K266	139.6	Anti dimer (1.03)	Tetramer (1.02)	30.7/11.3	138.0	Tetramer	3Q8T^44^	
TRIM25 (*H. sapiens*)	A194–Q356	164.0	Tetramer (1.15)	Para dimer (1.03)	38.4/18.8	119.3	Tetramer	4CFG^***^	
TRIM5 (*M. mulatta*)	M133–L241	169.8	Trimer (1.16)	Tetramer (1.14)	32.6/13.2	—	Aggregation	4TN3 (ref. 52)	
Prediction									
NDP52 (*H. sapiens*)	E141–E334	—	Para dimer (1.24)	Tetramer (1.06)	42.6/23.2	102.5 (2.4)	Dimer	****	

Para and Anti, Parallel and Antiparallel, respectively. SspB, Stringent starvation protein B; GCN4, General control protein GCN4; APC, Adenomatous polyposis coli protein; Atg16, Autophagy protein 16; SCOC, Short coiled-coil protein; LRRFIP1, Leucine-rich repeat flightless-interacting protein 1; Ndel1, Nuclear distribution protein nudE-like 1; TPM1, Tropomyosin alpha-1 chain; ROCK1, Rho-associated coiled-coil containing protein kinase 1; MDV1, Mitochondrial division protein 1; Mfn1, Mitofusin-1; LMNA, Prelamin-A/C; BECN1, Beclin-1; TRIM25, E3 ubiquitin/ISG15 ligase tripartite motif-containing protein 25; TRIM5, Tripartite motif-containing protein 5; NDP52, Calcium-binding and coiled-coil domain-containing protein 2; ^*^Results of coiled-coil prediction server (LOGICOIL)^20^; ^**^SspB fusion proteins with His_6_ tags; ^***^no reference available; ****no PDB ID.
