# Supplementary material for: ACCORD: an assessment tool to determine the orientation of homodimeric coiled-coils
Source: Sci Rep. 2017 Mar 7;7:43318. doi: 10.1038/srep43318 (PMC5339707; doi:10.1038/srep43318)
Supplement: Supplementary Information [file srep43318-s1.pdf]

Supplementary Figures and Tables

ACCORD: an assessment tool to determine the orientation of homodimeric coiled-coils

Byeong-Won Kim[1,2], Yang Ouk Jung[2], Min Kyung Kim[2], Do Hoon Kwon[2], Si Hoon Park[2], Jun Hoe Kim[2], Yong-Boo Kuk[2], Sun-Joo Oh[2], Leehyeon Kim[2], Bong Heon Kim[2], Woo Seok Yang[2] & Hyun Kyu Song[1,2,a]

[1]Center for Molecular Dynamics and Spectroscopy, Institute of Basic Science, Seoul 02841, Korea

[2]Division of Life Sciences, Korea University, 145 Anam-ro, Seongbuk-gu, Seoul 02841, Korea

[a]Correspondence: Hyun Kyu Song, Division of Life Sciences, Korea University, 145 Anam-ro, Seongbuk-gu, Seoul 02841, Korea, Tel: 82-2-3290-3457, Fax: 82-2-3290-3628, E-mail: hksong@korea.ac.kr

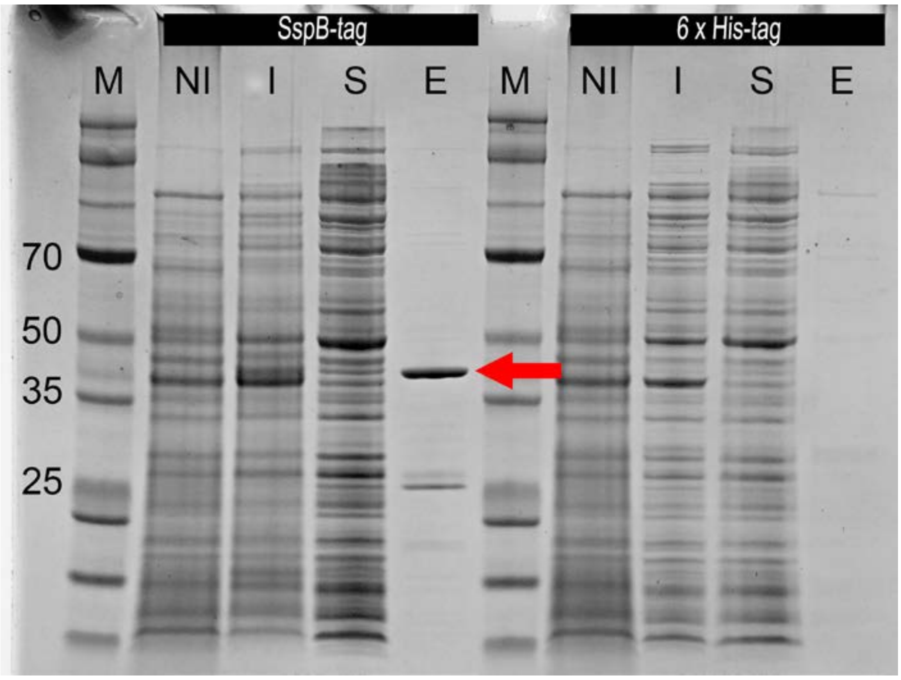

Supplementary Figure 1. Solubility test of the stringent starvation protein B (SspB)-fusion protein. Human ATG16L1 coiled-coil domain (residues 79 to 265) is fused with SspB and a hexa-histidine tag, respectively. The SspB-fused protein is marked with a red arrow. M, Molecular weight marker proteins, NI, non-induced total cell extract; I, induced total cell extract; S, soluble fraction; E, elution fraction from affinity chromatography.

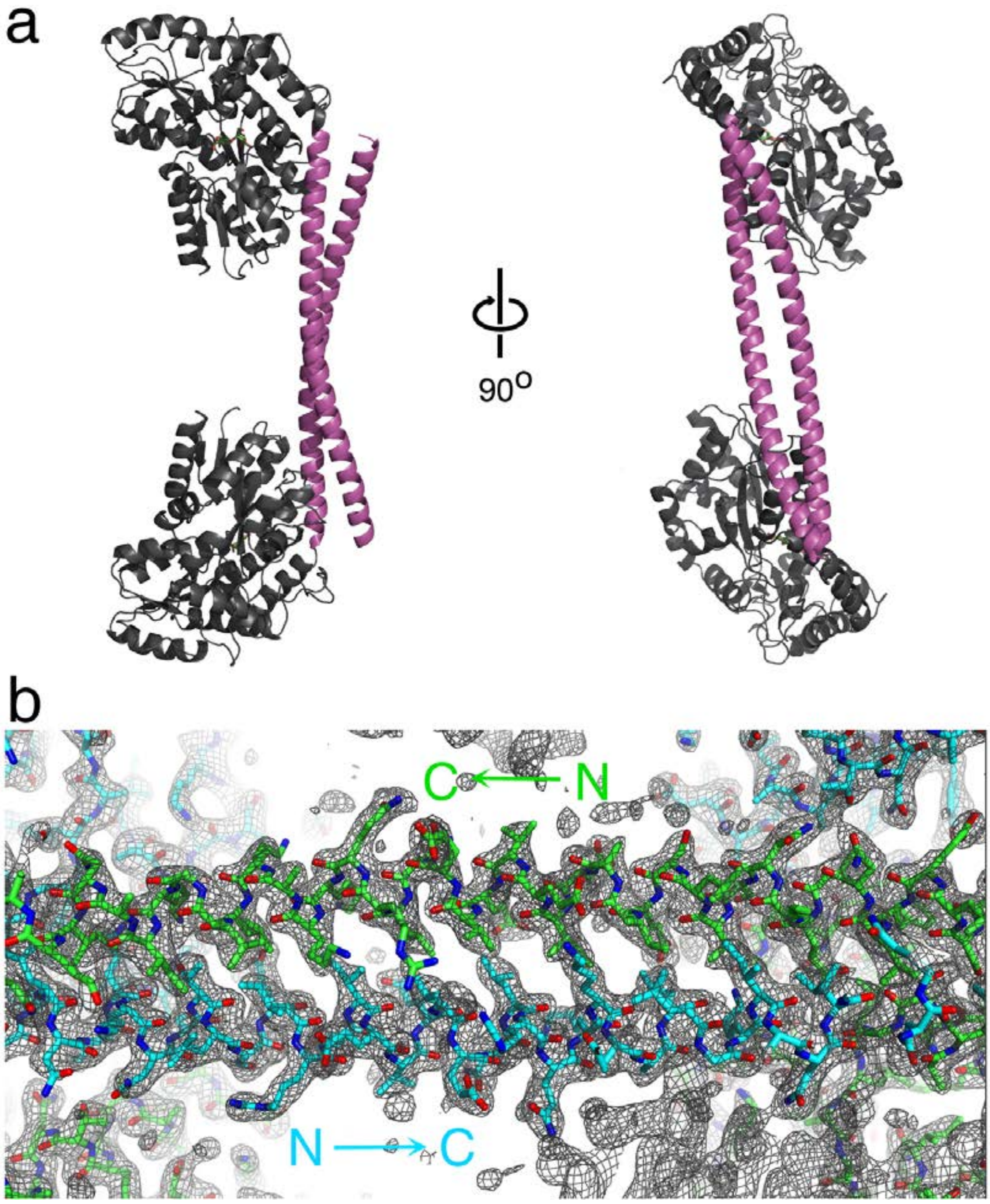

Supplementary Figure 2. Validation of the ACCORD results using X-ray crystallography. (**a**) Crystal structure of the MBP-MDV1 fusion protein. The MBP tag is located on the opposite site antiparallel to the coiled-coil (CC) of MDV1. (**b**) The final $2F_o$–F_c electron density map around the central CC. The map was calculated using 36.9–2.2 Å data and contoured at 1.0 σ. The directions of the CC chains are shown.

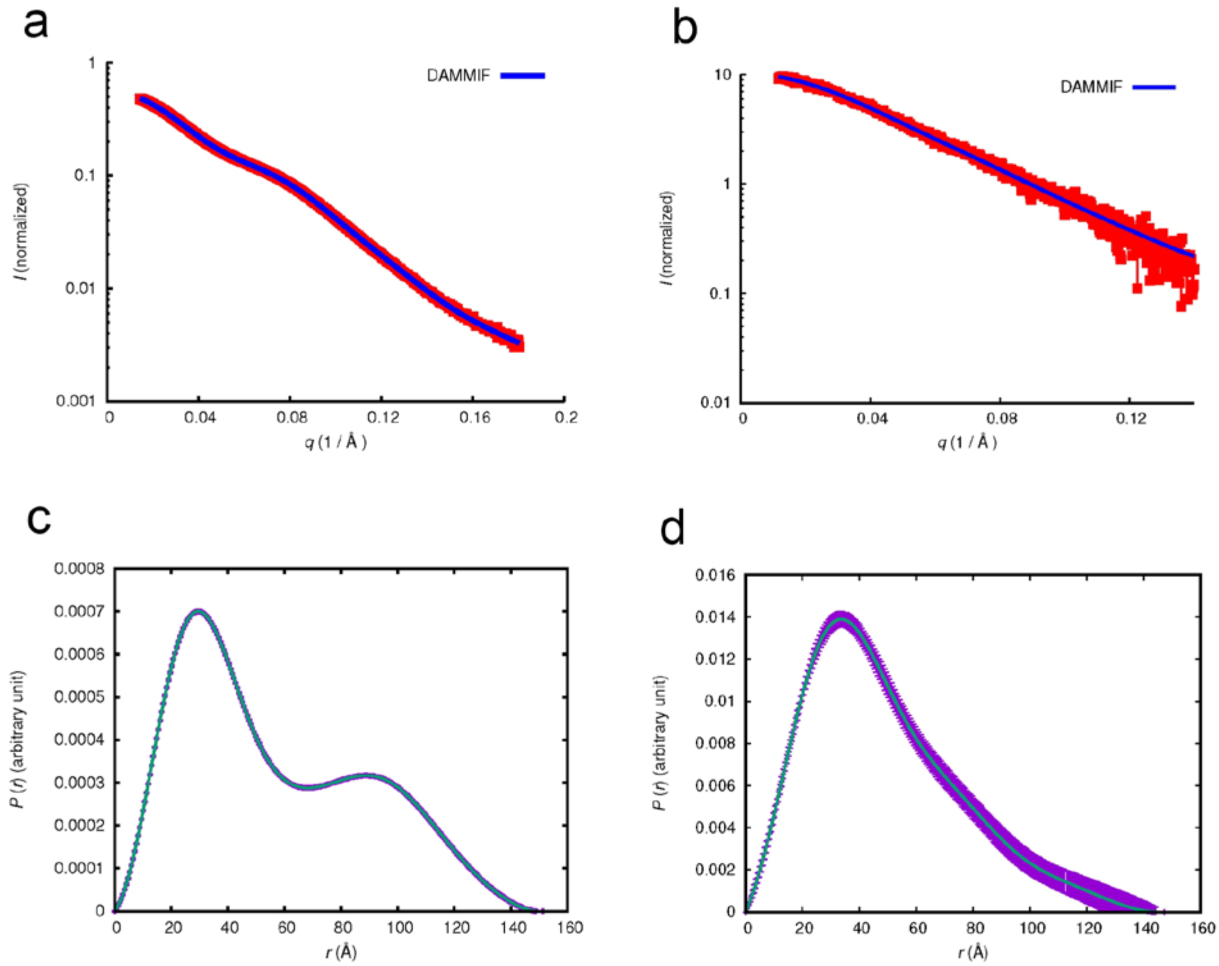

Supplementary Figure 3. Validation of the ACCORD results using SAXS experiments. The theoretical scattering curve calculated using DAMMIF (blue curve) fits well with the scattering intensity of MBP-MDV1 (red dots) (**a**) and MBP-NDP52 (red lines) (**b**). Distance distribution function, $P(r)$ (green line) with error bar (purple), of MBP-MDV1 (**c**) and the MBP-NDP52 fusion protein (**d**) showing D_{max} and R_g values.

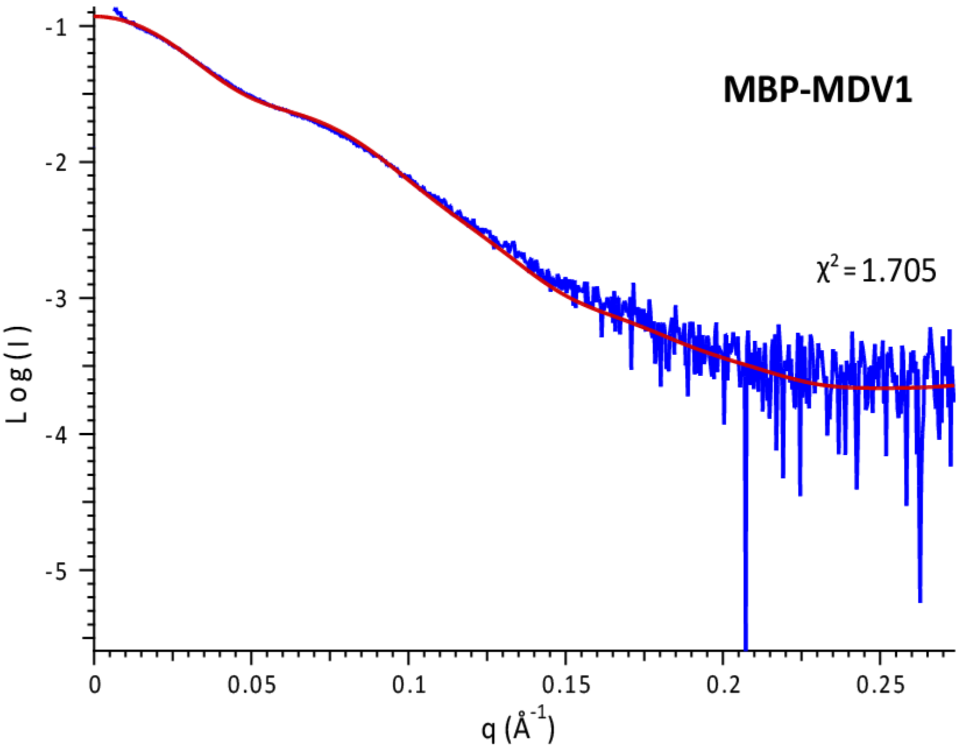

Supplementary Figure 4. Comparison of the experimental scattering intensity of MBP-MDV1 in solution (blue line) with the pattern calculated from the atomic coordinates of the crystal structure of MBP-MDV1 (red line) using the program CRYSOL. The experimental scattering data and the pattern calculated from the atomic coordinates match well.

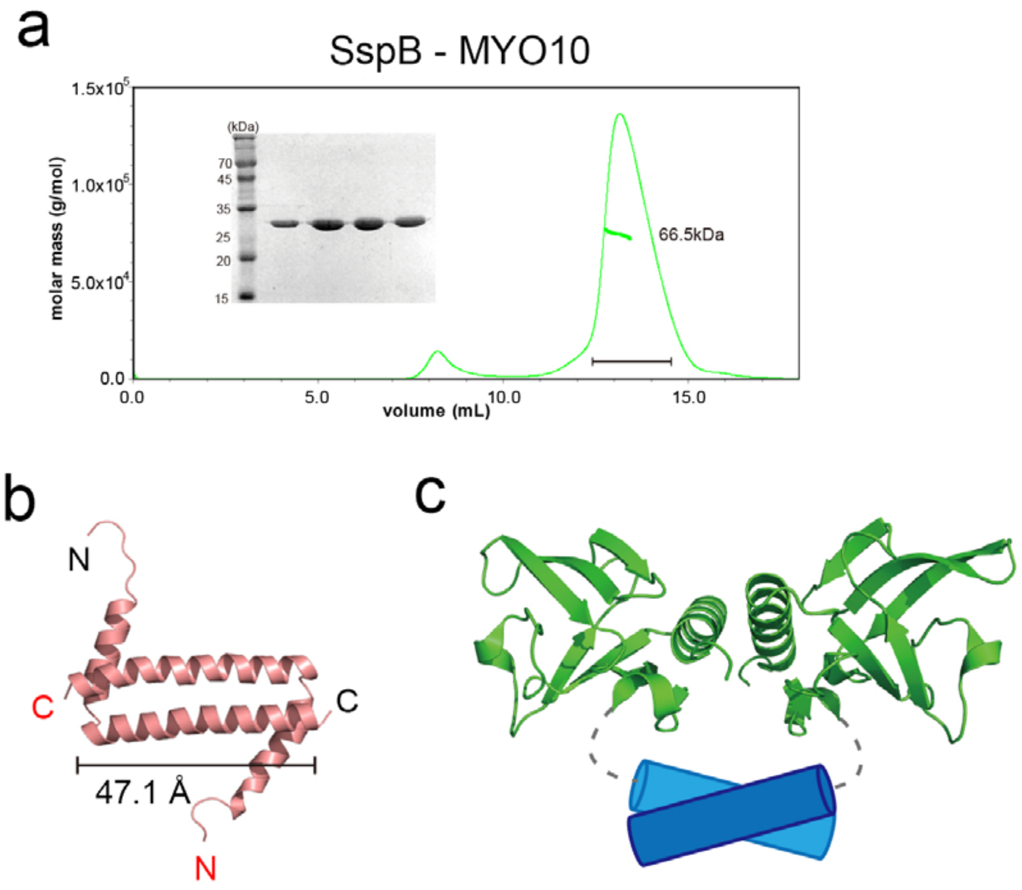

Supplementary Figure 5. ACCORD results with a short antiparallel coil-coil (CC). (**a**) SEC-MALS data of SspB-MYO10 showing a dimer in solution. Inset shows results of SDS-PAGE of the chromatography fractions. (**b**) Structure of antiparallel CC domain (CCD) of MYO10 (PDB ID: 2LW9). The length of the CCD is provided, and the N- and C-termini of each subunit are indicated. (**c**) Schematic model of the SspB-MYO10 structure. The antiparallel CCD of MYO10 fused with the SspB protein may form a dimer in this arrangement.

Supplementary Table 1. Data collection and refinement statistics

	MBP-MDV1
**Data collection**	
Space group	*C*2
Cell dimensions	
a, b, c (Å)	128.5, 102.9, 79.3
α, β, γ (°)	90.0, 102.5, 90.0
Resolution (Å)	36.9–2.2 (2.28–2.20)
R_{merge} (%)	7.2 (80.1)
Mean *I/σI*	20.67 (3.04)
Wilson B factor (Å^2)	50.07
Completeness (%)	99.1 (99.1)
Redundancy	35.9 (2.6)
**Refinement**	
Resolution (Å)	36.9–2.2 (2.28–2.20)
R_{work}/R_{free} (%)	22.5/28.4 (31.9/38.2)
No. atoms	
Protein	6,587
Ligand/ion	58
Water	194
Average B-factors (Å^2)	59.9
Protein	60.0
Ligand/ion	55.2
Water	56.2
R.m.s. deviations	
Bond lengths (Å)	0.009
Bond angles (°)	1.20
Ramachandran plot	
Favoured (%)	98
Outliers (%)	0

Values in parentheses are for the highest resolution shell.

Supplementary Table 2. SAXS data collection and analysis

	MBP-NDP52	MBP-MDV1
**Data collection parameters**		
Beamline	PAL 4C	PF BL10C
Beam geometry	0.1 mm × 0.3 mm	1.6 mm × 8 mm
Wavelength (Å)	0.7337	1.488
q range (Å^{-1})	0.001-0.16	0.0010-0.27
Exposure time (s)	10	10
Concentration range (mg ml^{-1})	2.6	9.8
Temperature (K)	283	289
**Structural parameters**		
$I(0)$ (cm^{-1}) [From $P(r)$]	17.55	0.56
R_g (Å) [From $P(r)$]	38.30	46.00
$I(0)$ (cm^{-1}) [From Guinier]	10.02	0.54
R_g (Å) [From Guinier]	36.46	42.97
D_{max} (Å)	147.26	151.42
Porod volume estimate, V_p (Å^3)	119,291.00	120,565.00
Dry volume calculated from sequence (Å^3)	59,595.89	58,835.16
**Molecular-mass determination**		
Molecular mass M_r [From Porod volume]	74,556.88	75,353.1
Calculated monomeric M_r from sequence	49,252.8	48,624.1
**Software employed**		
Primary data reduction	In-house program	FIT2D
Data processing	PRIMUS	
Ab initio analysis	DAMMIF	
Validation and averaging	DAMAVER	
Rigid-body modelling	Situs Program Suite	
Model representation	PyMOL/Chimera	

Supplementary Table 3. Sequences of SspB and coiled-coil proteins

	Name	Protein sequence
Tag	His$_6$-SspB	**MHHHHHHG***MDLSQLTPRRPYLLRAFYEWLLDNQLTPHLVVDVTLPGVQVPMEYARDGQIVLNIAPRAVGNLELANDEVRFNARFGGIPRQVSVPLAAVLAIYARENGAGTMFEPEAAYDEDTSIMNDEEASADNETVMSVIDGDKPDHDDDTHPDDEPPQPPRGGRPALRVVK**GS****
Parallel	GCN4	RMKQLEDKVEELLSKNYHLENEVARLKKLVGER
	APC	AAASYDQLLKQVEALKMENSNLRQELEDNSNHLTKLETEASNMKEVLKQLQGSI
	Atg16	VSHDDALLNTLAILQKELKSKEQEIRRLKEVIALKNKNTERLNDELISGTIENNVLQQKLSDLKKEHSQLVAR
	SCOC	MMNADMDAVDAENQVELEEKTRLINQVLELQHTLEDLSARVDAVKEENLKLKSENQVLGQYIENLMSASSVFQTTDTKSKRK
	LRRFIP1	DSLAEVEEKYKKAMVSNAQLDNEKTNFMYQVDTLKDMLLELEEQLAESRRQYEEKNKEFEREKHAHSILQFQFAEVKEALKQREEMLE
	Ndel1	DFSSLKEETAYWKELSLKYKQSFQEARDELVEFQEGSRELEAELEAQLVQAEQRNRDLQADNQRLKYEVEALKEKLEHQYAQSYKQVSVLED
	TPM1	MDAIKKKMQMLKLDKENALDRAEQAEADKKAAEERSKQLEDELVALQKKLKGTEDELDKYSESLKDAQEKLELADKKATDAESEVASLNRRIQLVEEE
	ROCK1	LANEKLSQLQKQLEEANDLLRTESDTAVRLRKSHTEMSKSISQLESLNRELQERNRILENSKSQTDKDYYQLQAILEAERRDRGHDSEMIGDLQARITSLQEEVKHLKHNLEKVEGERKEAQDMLNHSEKEKNNLEIDLNYKLKSLQQRLEQEVNEHKVTKARLTDKHQSIEEAK
Anti-parallel	MDV1	QTLVNSLEFLNIQKNSTLSEIRDIEVEVENLRQKKEKLLGKIANIEQNQLLLEDNLKQIDDRLDFLEEYG
	MYO10	ENKQVEEILRLEKEIEDLQRMKEQQELSLTEASLQKLQERRDQELRRLEEE
	Mfn1	FTSANCSHQVQQEMATTFARLCQQVDVTQKHLEEEIARLSKEIDQLEKIQNNSKLLRNKAVQLESELENFSKQFLH
	LMNA	ARERDTSRRLLAEKEREMAEMRARMQQQLDEYQELLDIKLALDMEIHAYRKLLEGEEERLRLSPSPTSQRS
	BECN1	EDDSEQLQMELKELALEEERLIQELEDVEKNRKIVAENLEKVQAEAERLDQEEAQYQREYSEFKRQQLELDDELKSVENQMRYAQTQLDKLKK
	TRIM25	ASADLEATLRHKLTVMYSQINGASRALDDVRNRQQDVRMTANRKVEQLQQEYTEMKALLDASETTSTRKIKEEEKRVNSKFDTIYQILLKKKSEIQTLKEEIEQSLTKRDEFEFLEKASKLRGISTKPVYIPEVELNHKLIKGIHQSTIDLKNELKQCIGRLQ
	TRIM5	MEEVAQEYHVKLQTALEMLRQKQQEAEKLEADIREEKASWKIQIDYDKTNVSADFEQLREILDWEESNELQNLEKEEEDILKSLTKSETEMVQQTQYMRELISELEHRL
Unknown	NDP52	EVEEIEQHNKELCKENQELKDSCISLQKQNSDMQAELQKKQEELETLQSINKKLELKVKEQKDYWETELLQLKEQNQKMSSENEKMGIRVDQLQAQLSTQEKEMEKLVQGDQDKTEQLEQLKKENDHLFLSLTEQRKDQKKLEQTVEQMKQNETTAMKKQQELMDENFDLSKRLSENEIICNALQRQKERLEGE

*His$_6$-tag, **BamHI restriction enzyme site
